# Bacteria Associated With Urinary Tract Infections, Antimicrobial Resistance Profiles, and Associated Factors Among Diabetes Mellitus Patients at Debre Tabor Comprehensive Specialized Hospital, Northwest Ethiopia

**DOI:** 10.1155/bmri/6664132

**Published:** 2026-04-10

**Authors:** Birhan Gebrie, Addisu Melese, Tewachew Awoke, Seble Worku, Adane Mihret, Awoke Derbie

**Affiliations:** ^1^ Department of Medical Laboratory Sciences, Tseda Health Science College, Gondar, Ethiopia; ^2^ Department of Medical Microbiology, Bahir Dar University, Bahir Dar, Ethiopia, bdu.edu.et; ^3^ Armauer Hansen Research Institute, Addis Ababa, Ethiopia, ahri.gov.et; ^4^ Department of Medical Laboratory Sciences, College of Medicine and Health Sciences, Debre Tabor University, Debre Tabor, Ethiopia, dtu.edu.et; ^5^ Department of Microbiology, Immunology, And Parasitology, School of Medicine, College of Health Sciences, Addis Ababa University, Addis Ababa, Ethiopia, aau.edu.et

**Keywords:** bacterial profile, Debre Tabor, diabetes mellitus, significant bacteriuria, UTI

## Abstract

**Background:**

Patients with diabetes mellitus are at increased risk of urinary tract infections (UTIs) due to impaired immune function and metabolic changes, which may lead to serious complications and increased healthcare burdens. In the catchment area of Debre Tabor, northwest Ethiopia, data on UTI‐causing bacteria and their antimicrobial resistance profiles among diabetic patients remain limited. Given the rising threat of antimicrobial resistance, continuous local surveillance is essential for effective management and antibiotic stewardship. This study is aimed at determining the bacterial etiologies associated with UTIs, their resistance profiles, and associated factors among diabetic patients at Debre Tabor Comprehensive Specialized Hospital.

**Methods:**

A cross‐sectional study was conducted from May 15, 2022 to July 13, 2022 among 246 diabetic patients who were recruited using convenience sampling technique. Sociodemographic and clinical data were collected using a structured questionnaire. Midstream urine samples were collected for bacterial isolation, identification, and antimicrobial susceptibility testing using standard bacteriological methods. Data were entered and analyzed using SPSS Version 25 software. Descriptive statistics were used to summarize the data, and binary logistic regression was performed to identify factors associated with UTIs. A p value <0.05 with a 95% confidence interval was considered statistically significant.

**Results:**

In this study, the overall prevalence of significant bacteriuria was 35/246 (14.2%). Of the 35 isolates, the majority 29 (82.9%) were Gram‐negative bacteria. The predominant isolate was Escherichia coli at 18 (51.4%). Gram‐negative isolates were resistant to amoxicillin, trimethoprim/sulfamethoxazole, and cefotaxime at rates of 57.1%, 39.3%, and 35.7%. The proportion of antibiotic resistance among Gram‐positive bacteria ranged from 33.3% to 100%. Enterococcus species showed resistance to all tested antimicrobials. The overall multidrug resistance rate was 25.7%. Having symptoms of UTI (AOR: 9.57, 95% CI: 2.27–40.35) and being female (AOR: 2.63, 95% CI: 1.01–6.84) were significantly associated with significant bacteriuria.

**Conclusion:**

A considerable prevalence of significant bacteriuria was observed. Moreover, bacterial isolates demonstrated moderately high resistance to commonly used antimicrobials. Regular monitoring of antimicrobial resistance patterns and strengthening infection prevention strategies among diabetic patients in the study area are recommended.

## 1. Background

Diabetes mellitus (DM) is a metabolic disorder characterized by high blood glucose levels [1]. The prevalence of DM is rising worldwide and is becoming a severe public health problem, especially in developing countries [2, 3]. Since 2017, the number of individuals living with diabetes globally has been estimated at 451 million [1], increasing to 536.6 million by 2021, and this number is projected to reach 783.2 million by 2045. Approximately, 80.6% of people living with diabetes live in low‐ and middle‐income countries. Global diabetes‐related health expenditures were estimated at $966 billion in 2021 and are projected to reach $1054 billion by 2045 [4]. Diabetes and associated complications in Ethiopia are major causes of morbidity and mortality with consequential economic impact [5]. Over time, patients with diabetes may develop complications such as cystopathy, nephropathy, and renal papillary necrosis, which predispose them to urinary tract infections (UTIs) [6]. UTI is an infection caused by microorganisms that affects the kidneys, ureters, bladder, or urethra [7].

The incidence of UTIs is higher in patients with DM due to changes in the host defense mechanism, microvascular disease in the kidneys, and the presence of diabetic cystopathy [8]. Moreover, higher glucose concentration in the urine provides an excellent culture medium for bacterial proliferation [9]. DM has been shown to increase the frequency of recurrent UTIs by two‐ to threefold [10], as well as the rate of complicated UTIs, including acute pyelonephritis, emphysematous UTIs, and renal abscess formation [11]. Patients with DM incur above‐average UTI treatment costs due to more frequent infections, higher complication rates, and greater use of hospital care and advanced antibiotics [12].

The most frequent causative agents of UTIs are bacteria, particularly Gram‐negative species [9, 10, 13]. In most studies, the majority of bacterial UTIs were caused by the Gram‐negative bacterium *Escherichia coli* [6, 13, 14]. Other bacterial causes of UTI include *Staphylococcus saprophyticus, Klebsiella pneumoniae*, *Proteus mirabilis, Enterococcus faecalis,* Group B *Streptococcus, Pseudomonas aeruginosa*, and *Staphylococcus aureus* [13–15]. These bacteria associated with UTIs possess multiple virulence factors that enhance their ability to colonize and persist in the urinary tract. These include adhesins such as pili/fimbriae, biofilm formation, proteases, toxins, siderophore‐mediated iron acquisition systems, and urease activity [16]. Drug resistance in these pathogens is increasing through enhanced efflux, production of penicillin‐binding proteins, and the production of *β*‐lactamases or other resistance enzymes [16, 17].

In Ethiopia, comorbidity of diabetes and UTI represents a substantial and growing burden. According to a systematic review and meta‐analysis, the prevalence of UTIs among diabetic patients was estimated at 15.97% [18]. In resource‐limited settings, such as many regions of Ethiopia, routine urine culture and antimicrobial susceptibility testing are often not included in regular diabetic follow‐up care, resulting in predominantly empirical treatment regimens. This practice can facilitate the emergence and spread of antimicrobial‐resistant bacteria causing UTIs. Supporting this concern, a nationwide antimicrobial resistance analysis indicated that, according to the Ethiopian standard treatment guideline for UTI, almost all first‐ and second‐line antimicrobial agents showed relatively high levels of resistance among the most common causative pathogens [19].

Despite this, there is limited local data from Debre Tabor and surrounding areas on the spectrum of bacteria associated with UTIs, their antimicrobial susceptibility patterns, and the associated risk factors among diabetic patients. This represents a significant research gap and underscores the need for studies that provide evidence‐based guidance for the management of UTIs in diabetic populations in the study area.

## 2. Materials and Methods

### 2.1. Study Setting

**Figure 1:**
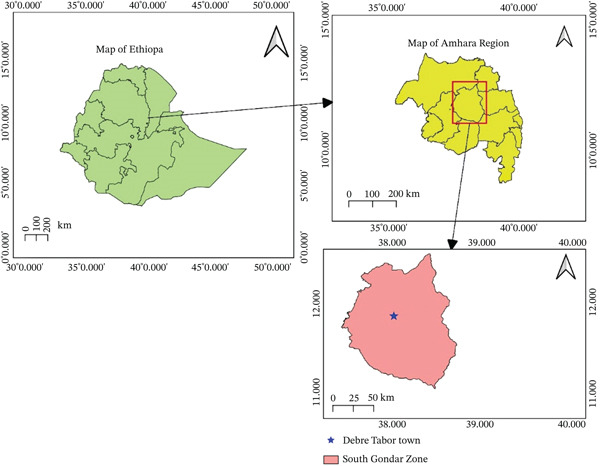
Map of the study area (Debre Tabor, Ethiopia).

A hospital‐based cross‐sectional study was conducted among DM patients in Debre Tabor Comprehensive Specialized Hospital (DTCSH), Northwest Ethiopia, from May 15 to July 13, 2022. Debre Tabor is located in the South Gondar Administrative Zone of the Amhara National Regional State, north central Ethiopia, about 100 km southeast of Gondar and 50 km east of Lake Tana. The surface area of Debre Tabor is about 31.87 km^2^. Topographically, the city is characterized by undulating terrain with significant elevation variation (Figure 1) [20]. According to the 2022 population projection, Debre Tabor town has a total population of 125,312, comprising 64,322 males and 60,990 females. During the period of data collection, about 2149 diabetic patients were receiving services from the hospital′s diabetic care program. The hospital follows the national treatment guidelines, whereby acute uncomplicated UTIs are primarily managed with nitrofurantoin or cotrimoxazole, with ciprofloxacin reserved as an alternative. Complicated UTIs, including many cases in patients with DM, are treated with ciprofloxacin as first‐line therapy, with ceftriaxone as an alternative. In severe cases or hospital‐acquired infections, broad‐spectrum agents such as meropenem are reserved for confirmed or suspected multidrug‐resistant (MDR) organisms, with alternatives such as piperacillin tazobactam; gentamicin may be added for synergy when necessary. All treatment shall ultimately be guided by culture and susceptibility results.

### 2.2. Source and Study Population

#### 2.2.1. Source Population

The source population comprised all diabetic patients who attended DTCSH.

#### 2.2.2. Study Population

Diabetic patients who visited DTCSH during the data collection period and met the inclusion criteria were included in the study.

### 2.3. Inclusion and Exclusion Criteria

#### 2.3.1. Inclusion Criteria

All DM patients who came for follow‐up and those who were newly diagnosed in the hospital during the study period were included.

#### 2.3.2. Exclusion Criteria

DM patients who were under antibacterial treatment within the 2 weeks prior to the data collection, known anatomical or neurologic urinary tract abnormalities, and pregnant women with induced DM were excluded from the study.

### 2.4. Variables

UTI was the dependent variable, whereas age, sex, marital status, residence, educational status, occupation, previous history of UTI, presence of UTI symptoms, type of DM, duration of diabetes, and fasting blood glucose level were the independent variables.

### 2.5. Sample Size Determination and Sampling Technique

#### 2.5.1. Sample Size Determination

The sample size was calculated using a single population proportion formula (Cochran′s formula) based on the proportion of significant bacteriuria (SBU) of 11.6% among DM patients from a previous study in Dessie [21], with a 95% confidence interval (Z*α*/2 = 1.96), and a 4% margin of error. The calculation was performed using OpenEpi, Version 3, resulting in a total sample size of 246.

#### 2.5.2. Sampling Technique

Study participants were recruited using a convenience sampling technique, where all eligible DM patients who attended the DTCSH during the study period were included until the required sample size was achieved.

### 2.6. Data and Specimen Collection

Sociodemographic and clinical data of DM patients were collected using a well‐designed questionnaire during their visits to the DM clinic by trained nurses. The questionnaire was developed from relevant literature, prepared in English, translated into the local language Amharic, and then translated back into English to ensure consistency. DM patients were instructed to bring 10–15‐mL midstream urine in a well‐labeled, sterile, dry, wide‐necked, leak‐proof, and screw‐capped container. Specimens were processed within 2 h of collection [22].

### 2.7. Urine Culture, Isolation, and Identification

Using a calibrated wire loop, a volume of l uL, well‐mixed urine was inoculated into MacConkey agar (Oxoid, Basingstoke, United Kingdom), 5% sheep blood agar (HiMedia, Mumbai, India), mannitol salt agar (HiMedia, Mumbai, India), and bile esculin agar (HiMedia, Mumbai, India). Then, after incubation at 35°C–37°C for 24–48 h, colony counts of ≥ 10^5^CFU/mL were considered SBU [22, 23]. Isolates were identified using colony characteristics, Gram reaction, and a panel of biochemical tests. Gram‐negative bacteria were identified using biochemical tests namely triple sugar iron, oxidase, indole, citrate, urea, motility medium, lysine–iron agar, and Gram‐positive bacteria were identified using coagulase, catalase, bile esculin agar, and mannitol salt agar. All culture media, except mannitol salt agar from Sigma‐Aldrich (St. Louis, Missouri, United States), were obtained from HiMedia (Mumbai, India) [22, 24].

### 2.8. Antimicrobial Susceptibility Testing (AST)

The AST was performed using the Kirby–Bauer disc diffusion method on Mueller–Hinton agar (MHA), HiMedia, Mumbai, India following CLSI guidelines [25]. The inoculums were prepared from pure culture by taking three to five pure colonies with a sterile wire loop and transferred to a tube containing 5 mL of sterile normal saline. Then, we mixed it gently until a homogenous suspension was formed and adjusted to a 0.5 McFarland turbidity standard. A sterile cotton swab was used to spread the suspension evenly over the entire surface of MHA [22].

The following antimicrobial discs were tested against the isolates: nitrofurantoin (300 *μ*g), ciprofloxacin (5 *μ*g), amoxicillin (10 *μ*g), trimethoprim/sulfamethoxazole (1.25/23.75 *μ*g), gentamicin (10 *μ*g), tetracycline (30 *μ*g), chloramphenicol (30 *μ*g), ceftazdime (30 *μ*g), cefotaxime (30 *μ*g), cefepime (30 *μ*g), imipenem (10 *μ*g), and amoxicillin/clavulanate (20/10 *μ*g) were used for Gram‐negative bacteria. Penicillin (10 units), tetracycline (30 *μ*g), ampicillin (10 *μ*g), gentamicin (10 *μ*g), and trimethoprim/sulfamethoxazole (1.25/23.75 *μ*g) were used for Gram‐positive bacteria. All antibiotic discs were obtained from Oxoid (Basingstoke, United Kingdom) and HiMedia (Mumbai, India). The results were read and interpreted after 16–18 h of incubation at 35°C ± 2°C according to CLSI 2022 guidelines [25].

### 2.9. Quality Assurance

Standard operating procedures were strictly followed during specimen collection, transportation, and processing steps. Prior to the actual data collection, a pretest was conducted on 5% of the total sample size to ensure the validity of the questionnaire. Patients were given clear instructions on how to collect a midstream urine sample. After the preparation of culture media, sterility was checked by incubating 5% of the media overnight at 35°C–37°C. Moreover, reference bacterial strains *E. coli* ATCC 25922 and *S. aureus* ATCC 25923 were used to examine the performance of the culture medium and to check the potency of antimicrobial discs.

### 2.10. Data Analysis

Data were entered and analyzed using SPSS Version 25 software. Logistic regression analysis was employed to assess the association between dependent and independent variables. All independent variables with a *p* value ≤ 0.25 in the bivariate analysis were subjected to multivariable analysis. A *p* value ≤ 0.05 with a 95% confidence interval was considered statistically significant.

## 3. Results

### 3.1. Sociodemographic and Clinical Characteristics

In this study, 246 study participants were included. Of the total participants, 136 (55.3%) were males, and the age range of the participants was 4–92 years, with a mean age of 44.5 years. The majority, 174 (70.8%) of the participants, were married, and 133 (54.1%) had no formal education. Around half of the study participants, 117 (47.6%) were living in rural settings, and 103 (41.9%) were farmers.

Of the total study participants, 136 (55.3%) had Type II DM and 137 (55.7%) had DM for fewer than or equal to 5 years. There were 141 (57.3%) participants who had fasting blood glucose levels greater than 126 mg/dL. The majority of participants (89.8%) did not report signs and symptoms of UTI (Table 1).

**Table 1:** Sociodemographic and clinical characteristics of the study participants at DTCSH (*n* = 246).

Variables		Frequency	Percentage
Gender	Male	136	55.3
	Female	110	44.7
Age (years)	≤ 20	31	12.6
	21–37	62	25.2
	38–54	69	28.0
	55–71	72	29.3
	> 71	12	4.9
Marital status	Single	51	20.7
	Married	174	70.8
	Divorced/separated	7	2.8
	Widowed	14	5.7
Educational status	No formal education	133	54.1
	Primary school	48	19.5
	Secondary school	20	8.1
	College and above	45	18.3
Residence	Rural	117	47.6
	Urban	129	52.4
Occupational status	Farmer	103	41.9
	Unemployed	28	11.4
	Self‐employed	62	25.2
	Student	17	6.9
	Government employee	36	14.6
Type of DM	Type I DM	110	44.7
	Type II DM	136	55.3
Duration of DM	≤ 5 years	137	55.7
	> 5 years	109	44.3
Fasting blood glucose level	≤ 126	105	42.7
	> 126	141	57.3
Previous UTI history	Yes	26	11.6
	No	220	89.4
Symptom of UTI	Yes	25	89.8
	No	221	11.2

Abbreviations: DM, diabetic mellitus; UTI, urinary tract infection.

### 3.2. The Proportion of SBU and Type of the Isolates

**Figure 2:**
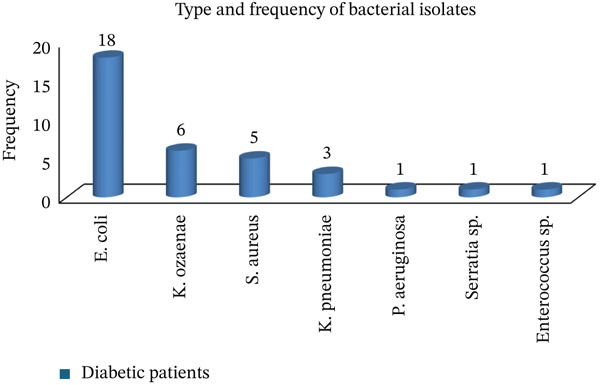
Type and frequency of bacterial isolates from urine specimens among DM patients at DTCSH.

The overall prevalence of SBU among DM patients was 35/246 (14.2%, 95% CI: 10.1%–19.2%). Out of the total 35 bacterial isolates, 29 (82.9%) were Gram‐negative. The predominant identified isolate was *E. coli* at 18 (51.4%) followed by *K. ozaenae* at 6 (17.1%), *S. aureus* at 5 (14.3%), and *K. pneumoniae* at 3 (8.6%) (Figure 2).

### 3.3. Antimicrobial Susceptibility Profile of the Isolates

The proportion of antibiotic resistance among Gram‐positive bacteria ranged from 33.3% to 100% as presented in Table 2. *S. aureus*, the most frequently isolated Gram‐positive bacteria, was resistant to penicillin. However, from the five *S. aureus* isolates, four (80%) were sensitive to gentamicin, whereas the antimicrobial resistance levels of the Gram‐negative isolates ranged from 3.6% to 57.1%. The degree of resistance to amoxicillin, trimethoprim–sulfamethoxazole, and cefotaxime, was at 57.1%, 39.3%, and 35.7%, respectively. Conversely, high rates of susceptibility of 96.4% and 86.2% were recorded for nitrofurantoin and cefepime, respectively.

**Table 2:** Antimicrobial susceptibility patterns of Gram‐positive isolates among DM patients at DTCSH.

Isolates (*n*)	Ptn	Antimicrobial agents, n (%)				
		TET	SXT	GN	AMP	PEN
*S. aureus* (five)	S	1 (20)	3 (60)	4 (80)	2 (40)	0 (0.0)
	R	4 (80)	2 (40)	1 (20)	3 (60)	5 (100)
*Enterococcus* sp. (one)	S	0 (0.0)	0 (0.0)	0 (0.0)	0 (0.0)	0 (0.0)
	R	1 (100)	1 (100)	1 (100)	1 (100)	1 (100)
Total (six)	S	1 (16.7)	3 (50)	4 (66.7)	2 (33.3)	0 (0.0)
	R	5 (83.3)	3 (50)	2 (33.3)	4 (66.7)	6 (100)

Abbreviations: AMP, ampicillin; GN, gentamicin; PEN, penicillin; Ptn, pattern; SXT, trimethoprim–sulfamethoxazole; TET, tetracycline.

All *E. coli* isolates were found to be sensitive to nitrofurantoin. However, these isolates exhibited a high level of resistance to amoxicillin in 12 (66.7%), trimethoprim–sulfamethoxazole in 8 (44.4%), amoxicillin–clavulanic acid in 6 (33.3%), and tetracycline in 5 (27.8%). Similarly, all *K. pneumoniae* isolates were found to be susceptible to nitrofurantoin, gentamicin, ceftazidime, cefepime, imipenem, ciprofloxacin, and tetracycline (Table 3).

**Table 3:** Antimicrobial susceptibility patterns of Gram‐negative isolates among DM patients at DTCSH.

Isolates (*n*)	Ptn	Antimicrobial agents, n (%)											
		NIT	CIP	AML	TET	C	SXT	GN	CTX	CAZ	FEP	AMC	IMP
*E. coli* (18)	S	18 (100)	16 (88.9)	6 (33.3)	13 (72.2)	14 (77.8)	10 (55.6)	17 (94.4)	13 (72.2)	15 (83.3)	16 (88.9)	12 (66.7)	16 (88.9)
	R	0 (0.0)	2 (11.1)	12 (66.7)	5 (27.8)	4 (22.2)	8 (44.4)	1 (5.6)	5 (27.8)	3 (16.7)	2 (11.1)	6 (33.3)	2 (11.1)
*K. pneumoniae* (3)	S	3 (100)	3 (100)	1 (33.3)	3 (100)	2 (66.7)	1 (33.3)	3 (100)	1 (33.3)	1 (33.3)	3 (100)	2 (66.7)	3 (100)
	R	0 (0.0)	0 (0.0)	2 (66.7)	0 (0.0)	1 (33.3)	2 (66.7)	0 (0.0)	2 (66.7)	2 (66.7)	00 (0.0)	1 (33.3)	0 (0.0)
*K. ozaenae* (6)	S	5 (83.3)	5 (83.3)	4 (66.7)	2 (33.3)	4 (66.7)	5 (83.3)	4 (66.7)	4 (66.7)	3 (50)	4 (66.7)	4 (66.7)	5 (83.3)
	R	1 (16.7)	1 (16.7)	2 (33.3)	4 (66.7)	2 (33.3)	1 (16.7)	2 (33.3)	2 (33.3)	3 (50)	2 (33.3)	2 (33.3)	1 (16.7)
*Serratia sp.* (1)	S	1 (100)	1 (100)	1 (100)	1 (100)	1 (100)	1 (100)	1 (100)	0 (0.0)	1 (100)	1 (100)	1 (100)	1 (100)
	R	0 (0.0)	0 (0.0)	0 (0.0)	0 (0.0)	0 (0.0)	0 (0.0)	0 (0.0)	1 (100)	0 (0.0)	0 (0.0)	0 (0.0)	0 (0.0)
*P.aeruginosa* (1)	S	—	1 (100)	—	—	—	—	1 (100)	—	—	1 (100)	1 (100)	1 (100)
	R	—	0 (0.0)	—	—	—	—	0 (0.0)	—	—	0 (0.0)	0 (0.0)	0 (0.0)
Total (29)	S	27 (96.4)	26 (89.7)	12 (42.9)	19 (67.9)	21 (75)	17 (60.7)	26 (89.7)	18 (64.3)	20 (71.4)	25 (86.2)	20 (69.0)	26 (89.7)
	R	1 (3.6)	3 (10.3)	16 (57.1)	9 (32.1)	7 (25)	11 (39.3)	3 (10.3)	10 (35.7)	8 (28.6)	4 (13.8)	9 (31.0)	3 (10.3)

Abbreviations: AMC, amoxicillin–clavunate; AML, amoxicillin; C, chloramphenicol; CAZ, ceftazdime; CIP, ciprofloxacin; CTX, cefotaxime; FEP, cefepime; GN, gentamicin; IMP, impenem; NIT, nitroforantion; SXT, trimethoprim–sulfamethoxazole; TET, tetracycline.

### 3.4. MDR Profile of the Isolates

Of the total isolates, 15 (42.9%) were found to be MDR. Of the MDR isolates, 11 (73.3%) were Gram‐negative. MDR is defined as a bacterium that is resistant to at least one agent in three or more antimicrobial categories according to Magiorakos et al. [26]. The MDR profile of Gram‐negatives as well as Gram‐positives is described in Table 4.

**Table 4:** Multidrug resistance profile of bacterial isolates among DM patients at DTCSH.

Gram‐negatives				
Combination of antimicrobials	Total n = 11 (%)	*E. coli* n = 7 (%)	*K. ozaenae* n = 3 (%)	*K. pneumoniae* n = 1 (%)
NIT, CIP, TET	1 (9.1)	—	1 (33.3)	—
SXT, IMP, AML	1 (9.1)	1 (14.3)	—	—
TET, CAZ, AMC	1 (9.1)		1 (33.3)	—
SXT, AMC, AML	3 (27.3)	3 (42.9)	—	—
AMC, AML, CTX	3 (27.3)	2 (28.6)	—	1 (100)
AML, AMC, FEP, CAZ, CTX, GN, SXT	1 (9.1)	—	1 (33.3)	—
CIP, TET, SXT, GN, CTX, CAZ, FEP, AML, AMC	1 (9.1)	1 (14.3)	—	—
Gram positives				
	**Total n = 4 (%)**	***S.aureus* n = 3 (%)**	***Enterococcus* sp. n = 1 (%)**	
AMP, SXT, PEN,	1 (25)	1 (33.3)	—	
TET, AMP, PEN	1 (25)	1 (33.3)		
SXT, TET, PEN	1 (25)	1 (33.3)	—	
AMP, TET, SXT, GN, PEN	1 (25)	—	1 (100)	

Abbreviations: AMC, amoxicillin–clavulanate; AML, amoxicillin; AMP, ampicillin; C, chloramphenicol; CAZ, ceftazidime; CIP, ciprofloxacin; CTX, cefotaxime; FEP, cefepime; GN, gentamicin; IMP, imipenem; PEN, penicillin; SXT, trimethoprim–sulfamethoxazole; TET, tetracycline.

### 3.5. Factors Associated With SBU

In bivariable analysis, sex (COR: 3.72, 95% CI: 1.69–8.11), marital status, educational status, presence of symptoms, occupational status, and history of previous UTI fulfilled the criteria to be included in multivariable logistic regression analysis. In the end, sex (AOR: 2.63, 95% CI: 1.01–6.84) and having symptoms of UTI (AOR: 9.57, 95% CI: 2.27–40.35) were statistically significantly associated with SBU (Table 5).

**Table 5:** Factors associated with SBU among diabetic patients at DTCSH.

Variables		SBU		COR (95% CI)	p value	AOR (95% CI)	p value
		Yes	No				
Sex	Male	10	126	1		1	
	Female	25	85	3.71 (1.69–8.11)	< 0.01	2.63 (1.01–6.84)	**0.048**
Age	4–20	3	28	1			
	21–37	7	55	1.19 (0.29–4.95)	0.81		
	38–54	12	55	1.97 (0.51–7.53)	0.32		
	55–71	12	60	1.87 (0.49–7.15)	0.36		
	72–92	1	11	0.85 (0.08–9.06)	0.89		
Marital status	Single	3	48	1		1	
	Married	30	144	3.33 (0.97–11.42)	0.06	2.29 (0.51–10.34)	0.279
	Divorced	1	6	2.67 (0.24–29.9)	0.43	1.05 (0.06–1.91)	0.974
	Widowed	1	13	1.23 (0.12–12.84)	0.86	0.33 (0.02–4.76)	0.415
Educational status	Diploma and above	4	41	1		1	
	Secondary school	1	19	0.54 (0.06–5.16)	0.59	0.35 (0.02–8.48)	0.521
	Primary school	6	42	1.46 (0.39–5.57)	0.58	1.75 (0.20–15.02)	0.609
	Cannot read and write	24	109	2.26 (0.74–6.90)	0.15	2.12 (0.24–18.91)	0.501
Residence	Urban	18	111	1			
	Rural	17	100	1.05 (0.51–2.15)	0.89		
Occupational status	Government employee	3	33	1		1	
	Unemployed	4	24	1.83 (0.38–8.96)	0.45	2.24 (0.17–29.61)	0.541
	Self‐employed	11	51	2.37 (0.62–9.15)	0.21	1.77 (0.15–20.81)	0.651
	Student	1	16	0.69 (0.07–7.14)	0.75	0.89 (0.06–59.09)	0.715
	Farmer	16	87	2.02 (0.55–7.40)	0.29	1.32 (0.10–18.91)	0.836
Type of DM	Type I DM	14	96	1			
	Type II DM	21	115	1.25 (0.6–2.6)	0.55		
Duration of DM	≤ 5 years	20	117	1			
	> 5 years	15	94	0.93 (0.45–1.92)	0.85		
FBS level (mg/dL)	≤ 126	15	90	1			
	> 126	20	121	0.99 (0.48–2.04)	0.98		
History of UTI	No	25	195	1		1	
	Yes	10	16	4.86 (2.00–11.91)	< 0.01	1.29 (0.29–5.79)	0.741
Symptoms of UTI	No	22	199	1		1	
	Yes	13	12	9.79 (3.99–24.1)	< 0.01	9.57 (2.27–40.35)	**0.002**

*Note:* 1, reference. Bolded data are statistically significant.

Abbreviations: AOR, adjusted odds ratio; COR, crude odds ratio; FBS, fasting blood sugar; UTI, urinary tract infection; SBU, significant bacteriuria.

## 4. Discussion

UTI is the most common bacterial infection in patients with DM. UTI caused by antimicrobial‐resistant bacterial strains in hospitals increases the cost of treatment, morbidity, and mortality among diabetics [9]. This study assessed the types of bacteria associated with UTIs, associated factors, and the resistance profile of these isolates to different classes of antimicrobials among diabetic patients.

According to this study, diabetes patients generally had a 14.2% (95% CI: 10.1%–19.2%) prevalence of SBU which was comparable to other similar studies conducted in Ethiopia, such as Dessie at 11.6% [21], Hawassa at 10.5% and 13.8% [27, 28], Metu at 16.7% [29], Gondar at 17.8% [30], Bule Hora at 15.7% [31], and Jigjiga at 10.5% [32]. It is also comparable with a study from China at 11.2% [33], and Romania at 12% [34]. The findings of this study revealed a low prevalence of SBU compared with two studies carried out in Addis Ababa, Ethiopia at 22.4% and 22.6% [35, 36], and Sudan at 19.5% [37]. Additionally, it is lower than reports from various regions of the world, such as a study in Iran at 32% [38], Kuwait at 35% [39], and Nepal at 50.7% [40]. Differences in patient characteristics, duration of diabetes, glycemic control, and access to routine screening may contribute to prevalence variation rather than environmental conditions alone.

In the current study, the proportion of SBU was more common in women (22.7%) than men (7.3%) diabetic patients. It is consistent with numerous other studies conducted in Ethiopia and elsewhere, where female diabetic patients had higher bacteriuria than males [13, 15, 27, 30, 40–43]. This aligns with the known influence of anatomical, hormonal, and behavioral factors that facilitate ascending infection in women. In diabetic women, the added effects of glucosuria, recurrent genital colonization, and reduced mucosal immunity further elevate the risk. These mechanisms provide plausible biological explanations for the sex difference observed in our study [44].

In this study, the major isolate was *E. coli* which was consistent with other studies in different parts of the world [13, 14, 30, 31, 37]. *E. coli* continues to dominate as a UTI‐causing bacterium in diabetic individuals because of its ability to express virulence determinants such as adhesins, fimbriae, and iron acquisition systems. In conditions of high glucose concentration, these virulence traits enhance bacterial growth, adherence, and persistence in the urinary tract [9, 44]. Moreover, in our study, from the total isolates, *S. aureus* accounted for 14.3%, which is comparable with other studies in Ethiopia that reported a proportion of 10.8%–18.2% [28, 29, 35].

Gram‐negative isolates showed a high level of susceptibility to nitrofurantoin (96.4%), ciprofloxacin (89.7%), and gentamicin (89.7%). Likewise, Worku et al. reported that Gram‐negative isolates were sensitive to nitrofurantoin at 100% and to gentamicin at 88.9% [13]. These results indicate that these drugs remain clinically useful options for empirical therapy in diabetic patients, especially where culture facilities are limited.

Similarly, in this study, Gram‐negative isolates showed 86.2% susceptibility to cefepime, a fourth‐generation cephalosporin which was consistent with a previous study by Mardhia et al., who also found that cefepime (92.3%) had better action towards Gram‐negative isolates [45]. Imipenem (89.7%) was also highly effective against Gram‐negative bacteria associated with UTIs; this result was similar to that of a study done in Nepal and Meerut City, India, where 91.9% and 84.5% of Gram‐negative bacteria associated with UTIs were sensitive to imipenem, respectively [40, 43]. In fact, carbapenems including imipenem are the last‐resort drugs for the treatment of severe infections or for patients suspected of harboring resistant bacteria [46]. These agents are valuable for complicated infections, but cautious use is recommended to prevent rapid resistance development. Relatively low susceptibility was observed to amoxicillin (42.9%), cefotaxime (64.3%), amoxicillin–clavulanic acid (69%), and trimethoprim–sulfamethoxazole (60.7%) than another study conducted in Dessie which reported 89.3%, 85.7%, and 81.8% susceptibility to cefotaxime, amoxicillin–clavulanic acid, and trimethoprim–sulfamethoxazole, respectively [21].

Of the total isolates, 42.9% were MDR, which was comparable with studies carried out in Dessie at 46.2% [21] and Bule Hora at 43.6% [31]. The increasing trend of MDR microorganisms has led to a multifaceted crisis in the world. The problem is challenging in developing countries because of the high prevalence of infection, unwise and irrational uses of antibiotics, and over‐the‐counter availability of antibiotics [47]. The MDR level observed emphasizes the clinical importance of culture‐guided therapy and the need to strengthen antimicrobial stewardship programs targeting diabetic patients.

As far as factors associated with SBU are concerned, females were 2.6 times more likely to develop SBU than men. This finding was consistent with a study from Metu and Bule Hora in Ethiopia, China, and Romania in which female diabetic patients had a higher likelihood of developing SBU or UTI than their male counterparts [29, 31, 33, 34]. This reinforces the need for sex‐specific preventive approaches such as education on hygiene, early symptom recognition, and regular screening in women with longstanding diabetes. Similarly, in the present study, those DM patients who had symptoms of UTI were 9.5 times more likely to develop SBU than those without symptoms. This was in line with a study conducted in Addis Ababa, Metu, and Jigjiga which found that diabetic individuals who had symptoms of UTI had a threefold higher risk of developing SBU than asymptomatic patients [13, 29, 32]. Clinically, this shows that symptoms remain a strong predictor of infection even in diabetic individuals who often have atypical presentations. This indicates symptom‐based screening as an efficient and cost‐effective approach in resource‐limited settings.

This study provides data on the prevalence and antimicrobial susceptibility of UTIs among diabetic patients in a comprehensive specialized hospital, which can guide empirical therapy in this setting. One limitation was the absence of HbA1c assessment. Additionally, molecular characterization of bacterial isolates and testing for anaerobic or difficult‐to‐culture pathogens were not performed, limiting understanding of resistance mechanisms and strain‐level epidemiology.

## 5. Conclusion and Recommendations

The overall prevalence of SBU among DM patients was found to be considerable. Gram‐negative isolates showed higher susceptibility to nitrofurantoin, gentamicin, ciprofloxacin, and imipenem while exhibiting high resistance to amoxicillin, trimethoprim–sulfamethoxazole, cefotaxime, and amoxicillin–clavulanate. Gram‐positive isolates demonstrated high resistance to penicillin and tetracycline. Female sex and the presence of urinary tract symptoms were significantly associated with SBU among DM patients.

Routine monitoring of antimicrobial resistance patterns is essential to guide appropriate antibiotic selection. Nitrofurantoin, ciprofloxacin, and gentamicin can be prioritized as empirical treatment options. Health education programs should be strengthened to raise awareness about UTI prevention, particularly among female diabetic patients. Integration of urine culture and susceptibility testing into routine diabetic follow‐up care is recommended to improve targeted management.

**Nomenclature**
AST: antimicrobial susceptibility testing
CLSI: Clinical and Laboratory Standards Institute
DM: diabetes mellitus
DTCSH: Debre Tabor Comprehensive Specialized Hospital
MDR: multidrug resistant
SBU: significant bacteriuria
UTI: urinary tract infection

## Author Contributions

Birhan Gebrie: conceived and designed the study, performed laboratory experiments, analyzed and interpreted the data, and wrote the first draft and final manuscript; Tewachew Awoke, Addisu Melese, Awoke Derbie, Seble Worku, and Adane Mhiret: conceived and designed the study, critically reviewed, and edited the final manuscript.

## Funding

This study was supported by the Bahir Dar University (10.13039/501100005872) and Armauer Hansen Research Institute (10.13039/100016237).

## Disclosure

All authors read and approved the manuscript. This article is a condensed and revised version of the author′s MSc thesis submitted to Bahir Dar University.

## Ethics Statement

This study was conducted in accordance with the principles of the Declaration of Helsinki. Ethical clearance was obtained from the Institutional Review Board (IRB) of the College of Medicine and Health Sciences of Bahir Dar University (Reference Number 386/2022). A written informed consent was obtained from each study participant. Bacteriologically positive results were communicated to the attending health professionals for better management of the study participants. All the data collected were kept confidential.

## Conflicts of Interest

The authors declare no conflicts of interest.

## Data Availability

The data that support the findings of this study are available from the corresponding author on reasonable request.
